# Pentraxin-3 as a predictive marker of mortality in sepsis: an updated systematic review and meta-analysis

**DOI:** 10.1186/s13054-022-04032-x

**Published:** 2022-06-08

**Authors:** Guobin Wang, Chunyan Jiang, Junjun Fang, Zhitao Li, Hongliu Cai

**Affiliations:** Department of Intensive Care Unit, The First Affiliated Hospital, Zhejiang University School of Medicine, No. 79 Qingchun Road, Hangzhou, 310003 Zhejiang People’s Republic of China

**Keywords:** Pentraxin-3, Sepsis, Mortality, AUC, Meta-analysis

## Abstract

**Background:**

The purpose of this study was to clarify the prognostic value of Pentraxin-3 (PTX3) on the mortality of patients with sepsis.

**Methods:**

Publications published up to January 2021 were retrieved from PubMed, EMBASE, and the Cochrane library. Data from eligible cohort and case–control studies were extracted for the meta-analysis. Multivariate regression analysis was used to evaluate the correlation of the outcomes with sample size and male proportion.

**Results:**

A total of 17 studies covering 3658 sepsis patients were included. PTX3 level was significantly higher in non-survivor compared to survivor patients (SMD (95% CI): −1.06 (−1.43, −0.69), *P* < 0.001). Increased PTX3 level was significantly associated with mortality (HR (95% CI): 2.09 (1.55, 2.81), *P* < 0.001). PTX3 showed good predictive capability for mortality (AUC:ES (95% CI): 0.73 (0.70, 0.77), *P* < 0.001). The outcome comparing PTX3 level in non-survivors vs. survivors and the outcome of the association between PTX3 and mortality were associated with sample size but not male proportion. AUC was associated with both sample size and male proportion.

**Conclusions:**

PTX3 level was significantly higher in non-survivor compared to survivor patients with sepsis. Elevated PTX3 level was significantly associated with mortality. Furthermore, the level of PTX3 might predict patient mortality.

## Background

Sepsis refers to the dysregulated host response against infection, leading to life-threatening organ dysfunction [1]. In 2017, there were approximately 48.9 million sepsis cases and 11 million related deaths worldwide, accounting for 19.7% of global deaths [2]. In recent years, the incidence and mortality of sepsis have been declining, but it is still the main cause of health damage worldwide [3]. Males with sepsis have worse clinical outcomes than females [4–6]. The occurrence and development of sepsis are closely related to systemic inflammation. Sepsis-related inflammatory markers play an important role in its clinical diagnosis, treatment evaluation, and organ function monitoring [7]. However, currently, C-reactive protein (CRP), calcitonin (PCT), and other inflammatory markers are more widely used for the diagnosis of sepsis and the prediction of its progression. Although these markers have a certain clinical diagnostic value for sepsis, their prognostic capabilities are relatively limited [7, 8].

Pentraxin-3 (PTX3) is an acute-phase protein belonging to the long-chain pentameric protein superfamily. As a key component of the human innate immune system, it plays an important role in the regulation of inflammation. In recent years, PTX3 has emerged as a promising biomarker for sepsis [9]. It has been demonstrated that the level of PTX3 is elevated in patients with sepsis. However, its predictive value for mortality remains controversial. The meta-analysis by Lee et al. showed that the elevated level of PTX3 was associated with an increased risk of patient mortality with sepsis [10]. More recently, several related studies have been published [9–14]. We aimed to provide an updated meta-analysis to further understand the predictive value of PTX3 in sepsis-related mortality.

## Methods

According to the Preferred Reporting Items for Systematic Reviews and Meta-Analyses (PRISMA) guideline [15], we performed this systematic review and meta-analysis. We started with searching relevant articles by the PICOS principle [16], followed by screening the publications on the basis of inclusion and exclusion criteria. Extracted data, including basic characteristics and outcomes, were reviewed by two different investigators (Guobin Wang and Chunyan Jiang) according to a pre-specified protocol.

### Eligibility criteria

The inclusion criteria included: (1) study population: adult patients with sepsis; (2) exposure: level of PTX3; (3) outcome: comparison of PTX3 level between non-survivors and survivors, the association between PTX3 and risk of mortality, and the prognostic value of PTX3 in mortality; (4) studies with hazard ratios (HR) or odds ratio (ORs) and area under the curve (AUC) data; and (5) language restricted to English.

### Search strategy

The databases of PubMed, Embase, and the Cochrane Library were systematically searched for eligible literature up to January 2021. During the search process, the MeSH terms ‘Pentraxin-3’ and ‘sepsis,’ as well as relevant key words, were used.

### Data extraction

Data extracted included study characteristics, including authors, the country where the study was performed, study design, sex, sample size, and follow-up time. The outcome included the comparison of PTX3 levels between non-survivors and survivors, the association between PTX3 and risk of mortality, and the prognostic value of PTX3 in mortality. HR and AUC were also extracted. For HR, multivariate-adjusted hazard ratios were extracted and analyzed. When values from the multivariate analysis were not available, those from univariate analysis were used.

### Quality of evidence

At the end of our research, a total of 17 studies entered our final model. The quality of evidence of all articles was assessed independently by two authors (Junjun Fang and Zhitao Li) according to the NOS criteria for cohort study or case–control study (http://www.ohri.ca/programs/clinical_epidemiology/oxford.asp). Discrepancies in the assessment were resolved through discussion until a consensus was reached.

### Statistical analysis

All of the analyses were performed using the STATA SE 14.0 software (StataCorp, College Station, Texas, USA). Standard mean differences (SMD), HR, AUC, and corresponding 95% confidence intervals (CIs) were used to evaluate the outcomes.

The extracted original HR data were grouped and analyzed according to optimal cutoff, median cutoff, original measurement value, and log transformation. If only ORs were reported, they were considered HRs. Statistical heterogeneity among these studies was calculated by Cochran’s *Q* test and the *I*^2^ index (over 50%, and *P*_heterogeneity_ < 0.1, high heterogeneity). Results will be pooled using a random-effects model. A *P* value less than 0.05 was considered statistically different. Multivariate linear regression analysis was performed on SMD, HR, and AUC according to sample size and male sex ratio. The meta-analysis was performed using a random-effects model. A *P* value less than 0.05 was considered statistically different. The Begg and Egger’s test was used to assess publication bias through trimmed and filled funnel plots.

## Results

### Study selection and characteristics of included studies

**Fig. 1:**
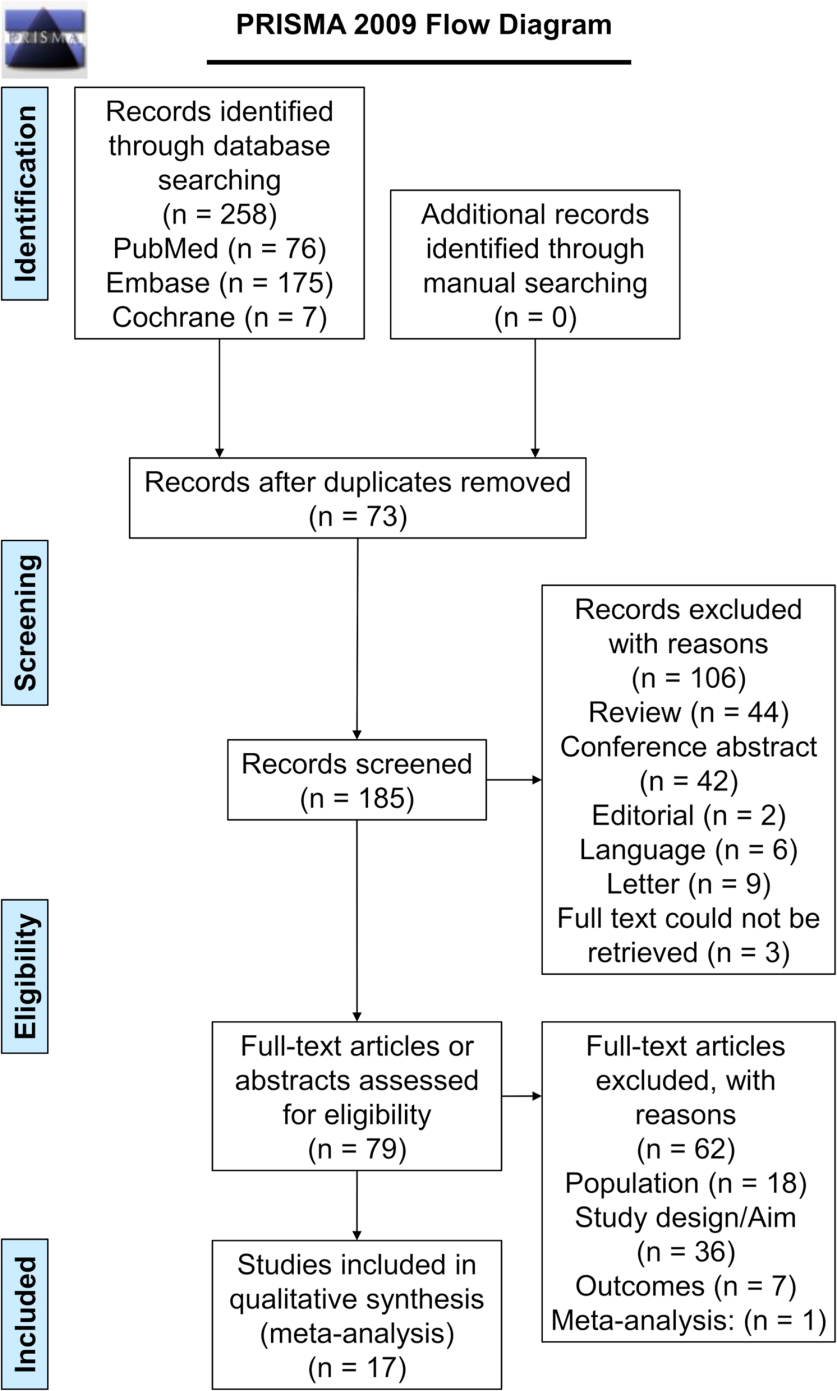
Flow diagram of the study selection process

The initial literature database search retrieved 258 relevant documents. After evaluating thoroughly, 17 studies that met the criteria were included in the final meta-analysis. The search process is illustrated in Fig. 1.

The characteristics of all included studies are summarized in Table 1. Seventeen eligible cohort and case–control studies covering 3658 patients with sepsis were included in this study. There was a substantial difference in the study populations in terms of microbial source of sepsis, infection site, and severity. The age of the patients varied from 18 to 100 years old. The follow-up time ranged from 10 to 873 days. Multivariate regression analyses on specific variables were reported in 8 studies. The NOS overall methodological score of all included studies revealed moderate to high quality (Tables 2 and 3).

**Table 1:** Literature search and characteristics of the included studies

Study, year	Country	Design	Population	Sample size (male)	Age, year	Follow-up, day	Outcome	Variables in the multivariate model
Muller, 2001	Switzerland	Prospective cohort study	critically ill patients (SIRS, sepsis, severe sepsis, septic shock)	101	–	–	OR, AUC	Univariate
Wagenaar, 2009	Indonesia	Prospective cohort study	severe leptospirosis	52 (37)	45 (32–55)	14	OR, AUC	Univariate
Mauri, 2010	Italy	Prospective cohort study	severe sepsis and septic shock	90 (56)	61 ± 15	90	PTX3 level, AUC	Univariate
Huttunen, 2011	Finland	Prospective cohort study	bacteremia	132 (70)	62 (18–93)	30	OR, PTX3 level	Univariate
Bastrup-Birk, 2013	Denmark	Prospective cohort study	systemic inflammatory response syndrome	261 (139)	63 (18–88)	873	HR	Multivariate: age, gender
Lin, 2013	China	Prospective cohort study	ventilator-associated pneumonia	136 (80)	65 ± 3	28	HR, PTX3 level, AUC	Multivariate: age, history of COPD, SOFA score, PO2/FiO2, creatinine
Uusitalo-Seppa¨la¨, 2013	Finland	Prospective cohort study	suspected infection	537 (310)	64 (18–100)	365	OR, PTX3 level, AUC	ICU stay, hypotension, use of vasopressors, disseminated intravascular coagulation, decreased Glasgow Coma Scale, Sepsis + organ dysfunction, multi-organ failure
Hansen, 2016	Denmark	Prospective cohort study	necrotizing soft tissue infections	135 (84)	61 (52–69)	510	HR, AUC	Multivariate: age, sex, Simplified Acute Physiology Score II, and chronic disease
Fan, 2017	Taiwan, China	Case–control study	acute decompensated cirrhotic patients	108 (71)	57.6 ± 11	90	HR	Multivariate: no parameters provided
Jie, 2017	China	Prospective cohort study	ICU septic shock	112 (55)	58.9 ± 13.9	28	HR, PTX3 level	Multivariate: no parameters provided
Kim, 2017	Korea	Prospective cohort study	severe sepsis	83 (47)	71 (64–77)	28	HR, PTX3 level, AUC	Univariate
Albert Vega, 2018	France	Case control study	septic shock	30 (21)	65 (19–86)	10	PTX3 level	Univariate
Hu, 2018	China	Prospective cohort study	sepsis and septic shock	141 (86)	64 (33–78)	28	HR, PTX3 level, AUC	Multivariate: no parameters provided
Hansen, 2020	Denmark	Prospective cohort study	ICU patients	547 (282)	66 (57–75)	28	HR, PTX3 level, AUC	Multivariate: age, sex, chronic disease and immunosuppression
Martin, 2020	Spain	Prospective cohort study	ICU septic shock	75 (53)	64 (49–74)	–	OR, PTX3 level, AUC	Multivariate: age, sex, and immunosuppression
Song, 2020	Korea	Prospective cohort study	sepsis and septic shock	160 (90)	77 (67–83)	28	HR, PTX3 level, AUC	Multivariate: age, positive blood culture, CRP, septic shock
Vassalli, 2020	Italy	Retrospective cohort study	septic patients	958	–	90	HR	Multivariate: troponin T, NT-proBNP, treatment

**Table 2:** NOS criteria for quality of cohort studies

Study	Representativeness of the exposed cohort	Selection of the non-exposed cohort	Ascertainment of exposure	Demonstration that the outcome of interest was not present at the start of the study	Comparability of cohorts on the basis of the design or analysis	Assessment of outcome	Was follow-up long enough for outcomes to occur	Adequacy of follow-up of cohorts	Total quality scores
Muller, 2001	☆	☆	☆	☆	☆	☆	–	–	6
Wagenaar, 2009	☆	☆	☆	☆	☆	☆	☆	☆	8
Mauri, 2010	☆	☆	☆	☆	☆	☆	☆	☆	8
Huttunen, 2011	☆	☆	☆	☆	☆☆	☆	☆	☆	9
Bastrup-Birk, 2013	☆	☆	☆	☆	☆	☆	☆	☆	8
Lin, 2013	☆	☆	☆	☆	☆☆	☆	☆	☆	9
Uusitalo-Seppa¨la¨, 2013	☆	☆	☆	☆	☆	☆	☆	☆	8
Hansen, 2016	☆	☆	☆	☆	☆☆	☆	☆	☆	9
Jie, 2017	☆	☆	☆	☆	☆	☆	☆	☆	8
Kim, 2017	☆	☆	☆	☆	☆	☆	☆	☆	8
Hu, 2018	☆	☆	☆	☆	☆	☆	☆	☆	8
Hansen, 2020	☆	☆	☆	☆	☆	☆	☆	☆	8
Martin, 2020	☆	☆	☆	☆	☆	☆	–	–	6
Song, 2020	☆	☆	☆	☆	☆	☆	☆	☆	8
Vassalli, 2020	☆	☆	☆	☆	☆	☆	☆	☆	8

**Table 3:** NOS criteria for quality of case–control studies

Study	Is the case definition adequate?	Representativeness of the cases	Selection of controls	Definition of controls	Comparability of cases and controls on the basis of the design or analysis	Ascertainment of intervention	The same method of ascertainment for cases and controls	Non-response rate	Total quality scores
Fan, 2017	☆	☆	–	☆	☆	☆	☆	–	6
Albert Vega, 2018	☆	☆	–	☆	–	☆	☆	–	5

### Comparing PTX3 levels between non-survivors and survivors

**Fig. 2:**
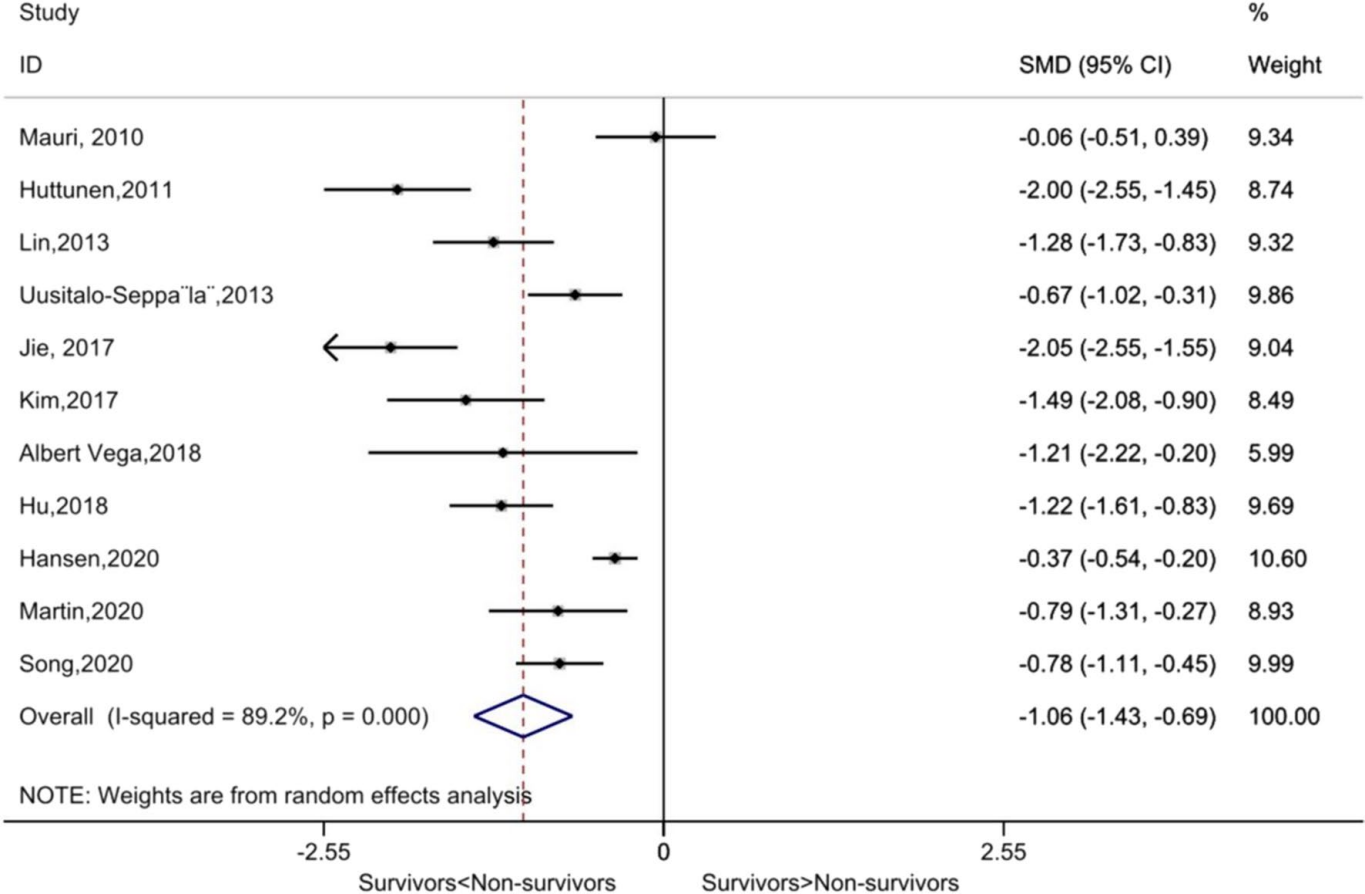
Forest plot of the levels of Pentraxin-3 (PTX3) between non-survivors and survivors in patients with sepsis

All 11 included studies reported significantly elevated circulating PTX3 concentrations in non-survivors compared to survivors. The pooled results consistently suggested that the levels of PTX3 in non-survivor patients were significantly higher than in survivor patients (SMD (95% CI): −1.06 (−1.43, −0.69), *P* < 0.001). The high *I*^2^ value of 89.2% (*P*_heterogeneity_ = 0.000) indicated the presence of significant statistical heterogeneity (Fig. 2).

### Association between Pentraxin-3 (PTX3) and mortality

**Fig. 3:**
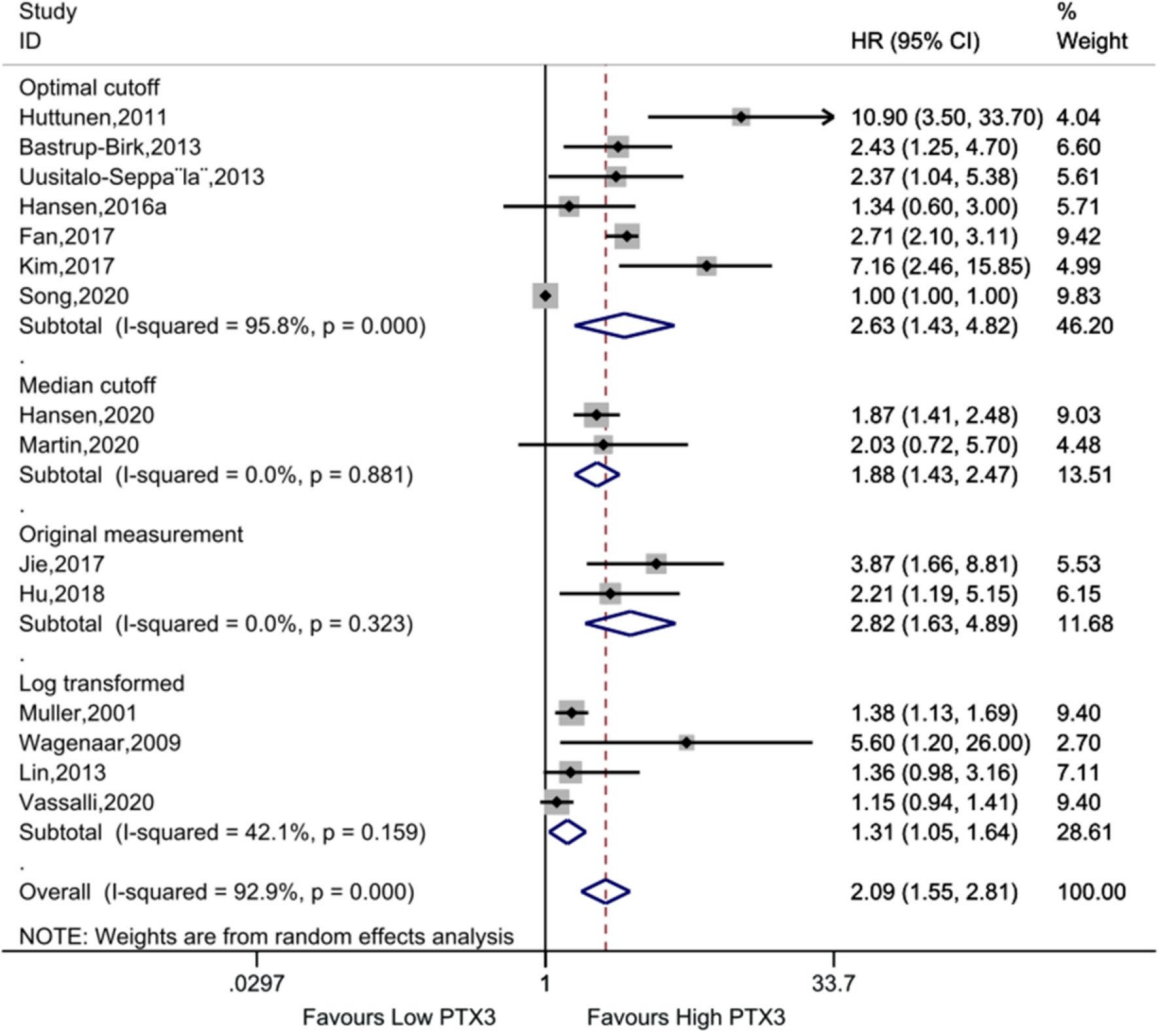
Forest plot of the association between Pentraxin-3 (PTX3) and mortality in patients with sepsis

Fifteen studies were included to assess the association between PTX3 level and the survival rate of patients. Pooled analysis showed that elevated PTX3 level was significantly associated with patient mortality (HR (95% CI): 2.09 (1.55, 2.81), *P* < 0.001) (Fig. 3). Substantial heterogeneity was detected in the overall analysis (*I*^2^ = 92.9%, *P*_heterogeneity_ = 0.000) and in the analysis based on the optimal cutoff data (*I*^2^ = 95.8%, *P*_heterogeneity_ = 0.000). Moderate heterogeneity was observed in the analysis according to the log transformation data (*I*^2^ = 42.1%, *P*_heterogeneity_ = 0.159). When the data were analyzed by the median cutoff and the original measurement value, no heterogeneity was found (both *I*^2^ = 0.0%).

### Predictive performance of PTX3 for mortality

**Fig. 4:**
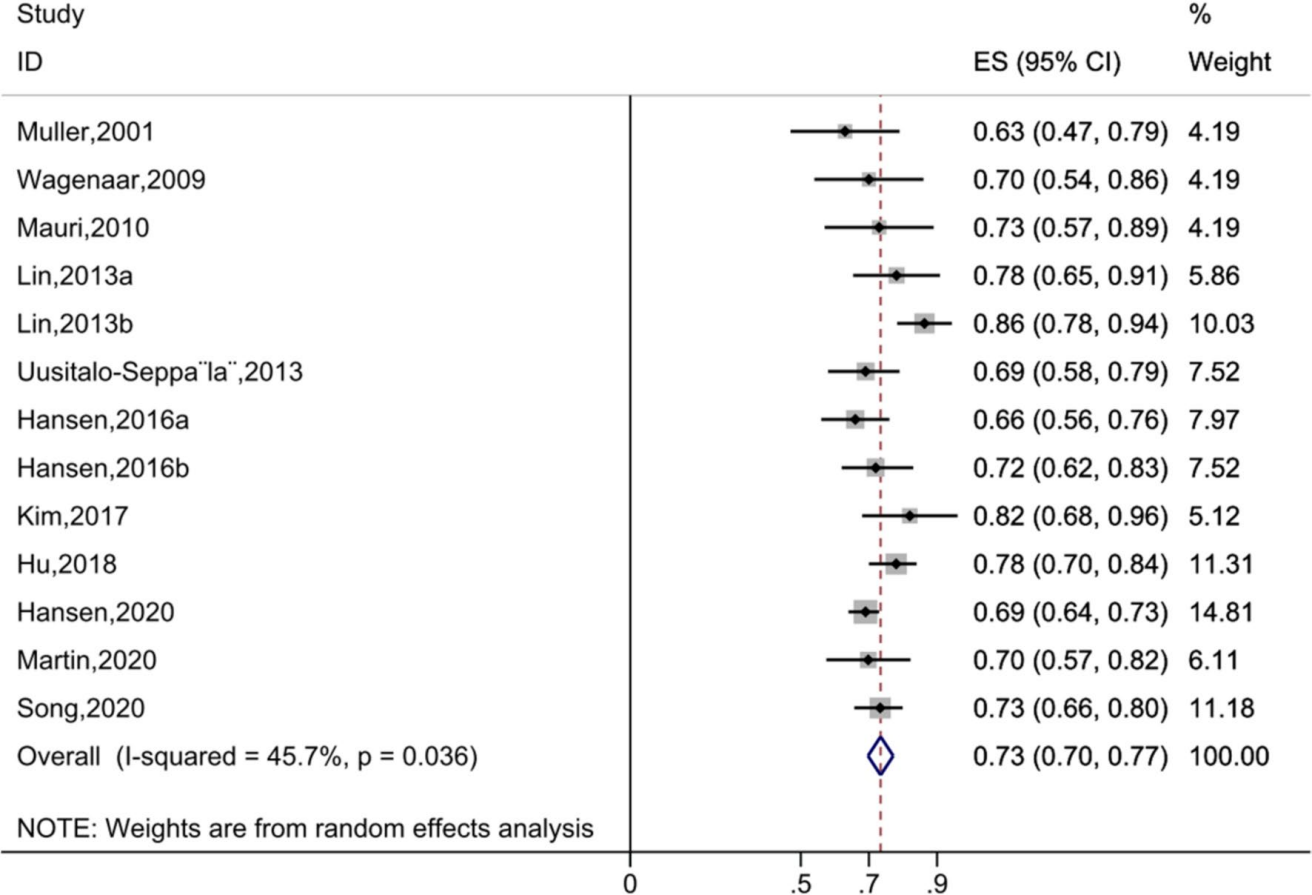
Forest plot of the predictive performance of Pentraxin-3 (PTX3) for mortality in patients with sepsis

Receiver operating characteristic (ROC) curves were used to determine the prognostic value of PTX3 for patients’ survival outcomes in 13 included studies. The pooled analysis revealed an overall AUC value of 0.73 (ES (95% CI): 0.73 (0.70, 0.77), *P* < 0.001), which suggested a good predictive ability of PTX3 for mortality (Fig. 4). The value of *I*^2^ was 45.7% (*P*_heterogeneity_ = 0.036), indicating moderate statistical heterogeneity.

### Multivariate regression analysis

**Fig. 5:**
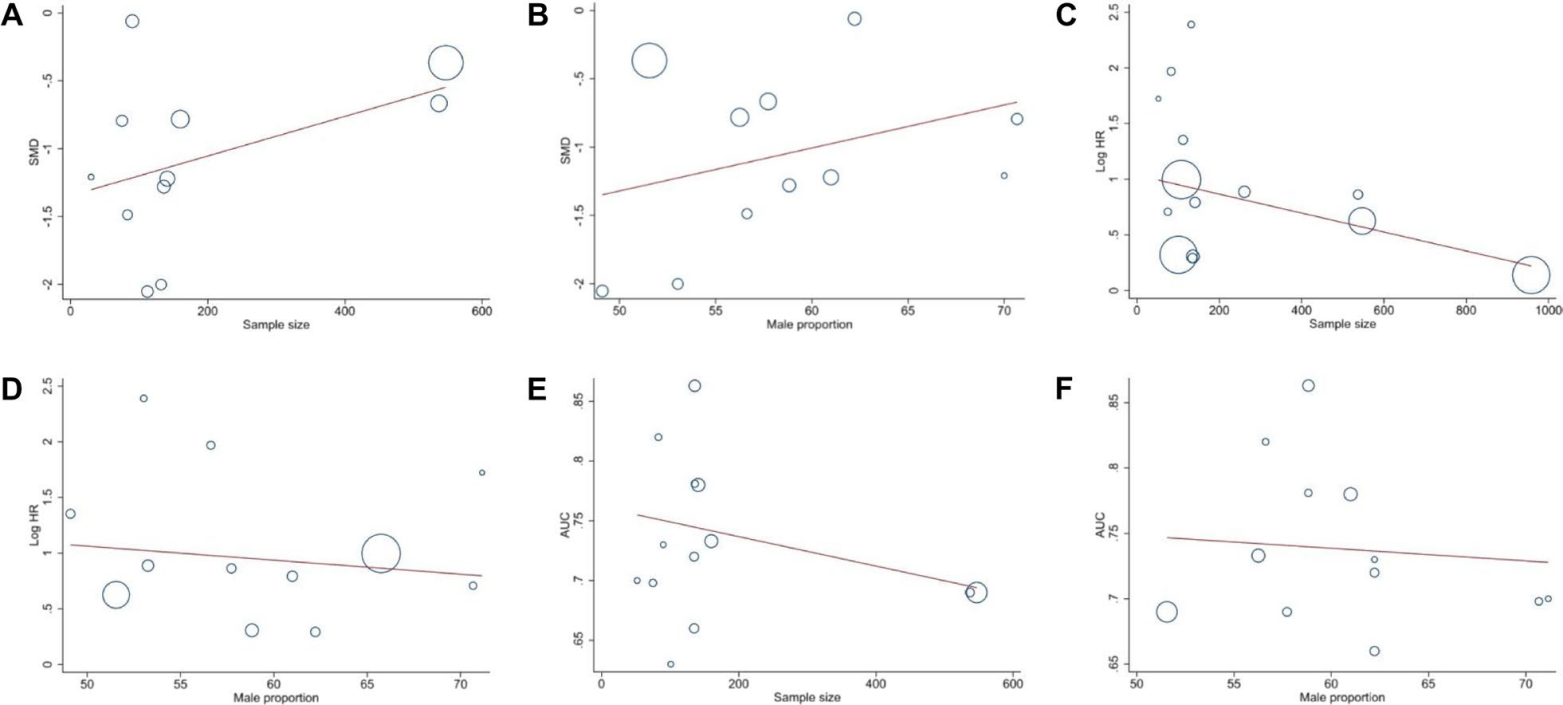
Linear regression of analyses of SMD, log HR, and AUC with the proportions of males and sample size in the included studies. **A** Linear regression of SMD sample size. **B** Linear regression of SMD by male proportion. **C** Linear regression of Log HR by sample size. **D** Linear regression of Log HR by male proportion. **E** Linear regression of AUC by sample size. **F** Linear regression of AUC by male proportion

Linear regression analysis suggested that SMD were associated with sample size (coefficient: −1.34547; *P* = 0.001), but not associated with male proportion (coefficient: −2.893843; *P* = 0.141) (Fig. 5A, B). The pooled regression analysis also showed that HR was correlated with sample size (coefficient: 1.036751; *P* < 0.001), but not with male proportion (coefficient: 1.691289; *P* = 0.313) (Fig. 5C, D). Moreover, AUC was revealed to be related to sample size (coefficient: 0.7613941; *P* < 0.001) and male proportion (coefficient: 0.7962401; *P* = 0.007) (Fig. 5E, F).

### Sensitivity analysis and publication bias

**Fig. 6:**
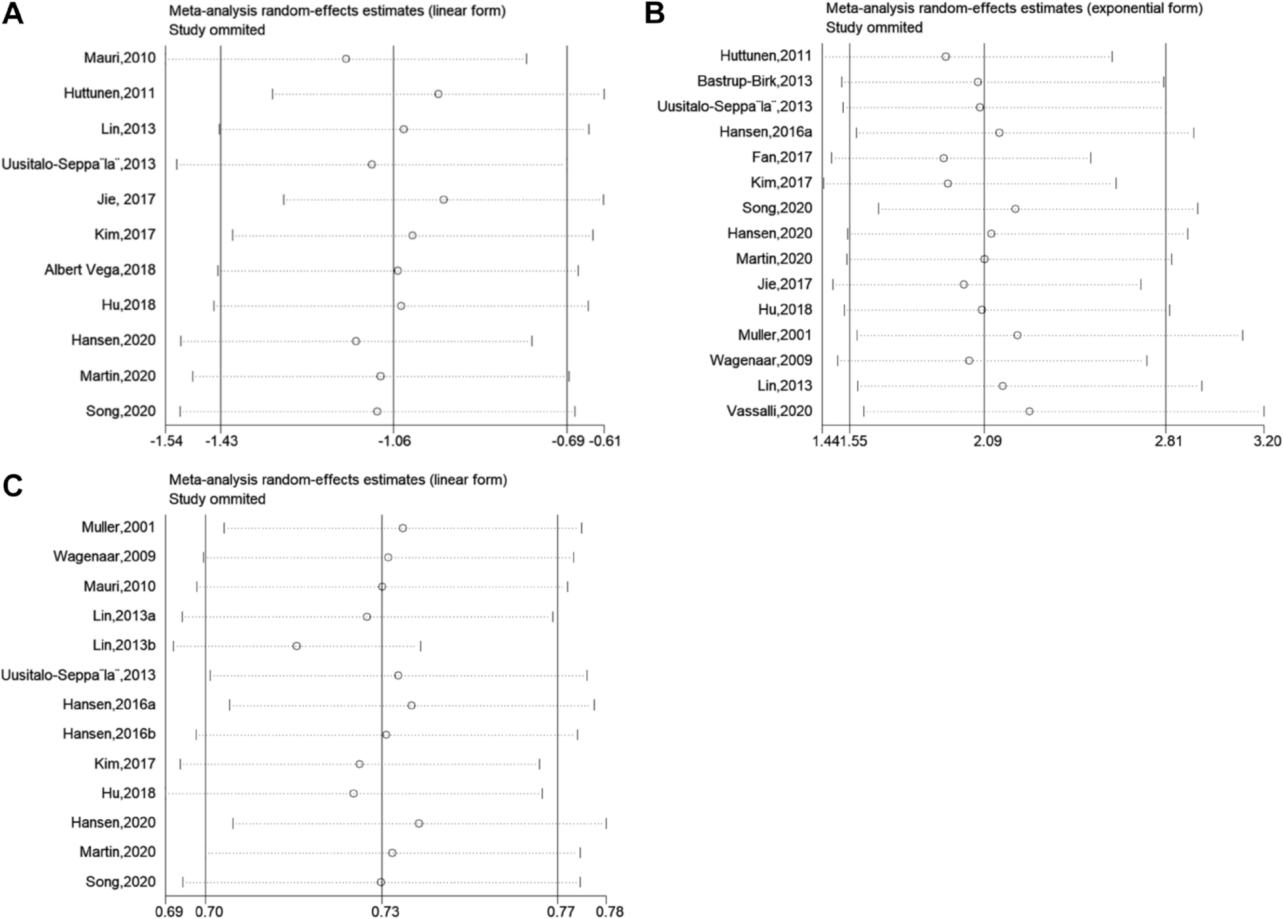
Sensitivity analyses. **A** Sensitivity analysis of SMD. **B** Sensitivity analysis of HR. **C** Sensitivity analysis of AUC

**Fig. 7:**
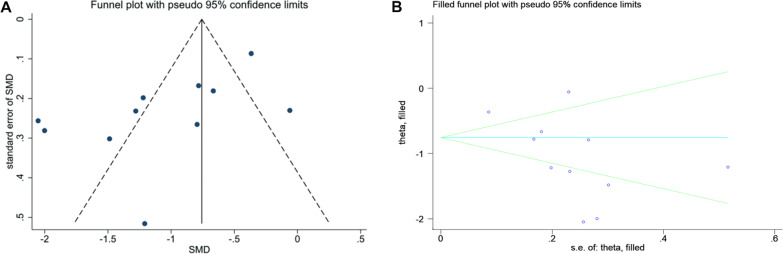
**A** Funnel plot of the standard error of SMD by SMD. **B** Filled funnel plot of the standard error of SMD by SMD

**Fig. 8:**
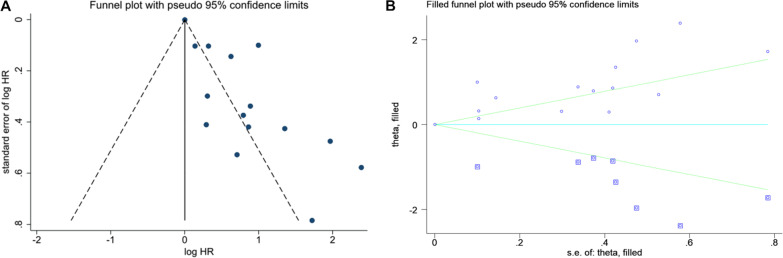
**A** Funnel plot of the standard error of log HR by log HR. **B** Filled funnel plot of the standard error of log HR by log HR

**Fig. 9:**
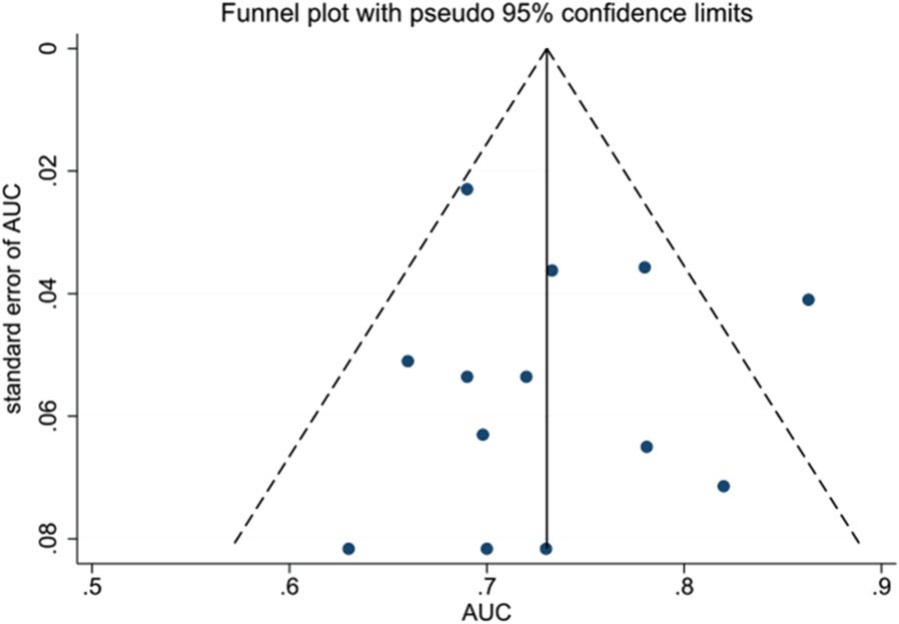
Funnel plot of the standard error of log AUC by log AUC

Sensitivity analysis demonstrated that our result was robust (Fig. 6A–C). The funnel plot of the standard error of SMD by SMD showed asymmetry, suggesting publication bias (Egger test: *P* = 0.020; Begg test: *P* = 0.119) (Fig. 7A). The filled funnel plot of the standard error of SMD by SMD consisting of 11 studies also revealed asymmetry (Fig. 7B). Significant asymmetry was observed in the funnel plot of the standard error of log HR by log HR (Egger test: *P* < 0.001; Begg test: *P* = 0.729) (Fig. 8A), whereas the filled funnel plot of the same parameter, including 23 studies, showed symmetrical distribution (Fig. 8B). In addition, the publication bias was found insignificant for the AUC outcome. The funnel plot of the standard error of log AUC by log AUC showed symmetry, and no statistically significant difference was observed (Fig. 9).

## Discussion

Our meta-analysis suggested that serum PTX3 levels in non-survivors with sepsis were higher than in survivor patients. Increased PTX3 level was significantly associated with patient mortality. The correlation analysis suggested that these outcomes were associated with sample size but not male proportion. Moreover, the ROC curve analysis indicated a good prognostic value of PTX3 in predicting mortality in patients with sepsis, and the AUC was related to both sample size and male proportion variables.

PTX3 plays an important role in regulating the innate immune mechanisms to prevent diseases, and it behaves as an acute-phase response protein [17]. Under normal physiological conditions, plasma PTX3 levels are low (< 2 ng/mL in humans) but increase rapidly during the course of inflammatory conditions, reaching 100–1,000 ng/ml depending on the severity of the disease [18]. Upregulated plasma PTX3 levels have been demonstrated in cardiovascular diseases, cancer, and infections [19–21]. PTX3 has been considered a biomarker of disease severity in different infections caused by bacteria, fungi, or viruses [22, 23]. Many studies have analyzed PTX3 plasma levels in patients with sepsis [24–34]. It is shown that the circulating levels of PTX3 exhibit a gradient from systemic inflammatory response syndrome (SIRS) to sepsis and septic shock in infection [32]. This evidence suggests that PTX3 is correlated with disease severity and degree of organ dysfunction [17]. In this meta-analysis, the pooled results from 11 studies found that the level of PTX3 was significantly higher in non-survivors of sepsis compared to survivors, which also indicates that survivors are lower values. In a previous meta-analysis, Lee et al. found that PTX3 was significantly higher in patients with more severe sepsis compared to those with less severe sepsis and higher in non-survivors compared to survivors, which was consistent with our results [35].

We further examined the relationship between PTX3 level and the risk of patient mortality. The pooled analysis suggested that high levels of PTX3 were significantly associated with mortality in patients with sepsis. Therefore, PTX3 level was linked with both disease severity and mortality in sepsis. Lee et al. also showed that elevated PTX3 increased patient mortality risk by  twofold [35]. Additionally, we performed multivariate regression analysis to explore the defined outcomes adjusted by variables of sample size and male proportion. The results suggested that the difference in PTX3 level in non-survivors vs. survivors and the correlation between PTX3 and mortality were associated with sample size but not associated with male proportion.

PTX3 level could be used for survival prediction of patients who developed sepsis, and the predictive value of PTX3 for sepsis has been performed by using ROC curve analysis. Song et al. showed that PTX3 at a cutoff level of 26.90 ng/mL provided a sensitivity of 88.9 (74.5–95.0) and a specificity of 49.5 (40.4–58.7) in predicting 28-day mortality in patients with sepsis, yielding an AUC of 0.734 (0.656–0.811) [13]. Similarly, according to Hu et al., when the serum PTX3 cutoff concentration was 49.90 ng/mL, the mortality prediction sensitivity was 83.3%, the specificity was 64.2%, and the AUC was 0.78 (0.70–0.84) in patients with sepsis and septic shock [12]. Another study by Kim et al. suggested that PTX3 had the largest AUC value for the prediction of 28-day all-cause mortality in severe septic patients compared to other biomarkers, including procalcitonin, delta neutrophil index, and CRP [29]. Our meta-analysis consistently suggested that serum PTX3 level was a useful prognostic biomarker for patients with sepsis. Furthermore, in this pooled study, multivariate logistic regression analysis indicated that the PTX3 predictive value of AUC was correlated with both sample size and male proportion.

This meta-analysis also had limitations. First, the sample size of this system study was still relatively small, and most included data were based on single-center studies. Second, there was high heterogeneity across studies, which might be due to variations in patient age, sex, ethnicity, follow-up time, disease pathogenesis, comorbidities, and severity. Third, in the ROC analysis, the cutoff value of PTX3 in predicting patient mortality varied greatly, and the optimal value was not determined. Furthermore, publication bias was detected, probably because the authors chose to publish positive results. Negative results are more difficult to publish, mainly because it can be difficult to determine whether a negative result is really due to the absence of an association or to a lack of statistical power that led to non-significant *P*-values [36, 37]. Still, carefully designed and analyzed studies that suggest negative results are important because negative results are still results, and they are important to assess the real association between a factor and an outcome [36, 37]. When publication bias is detected, there is a risk of overestimation of an effect, but that overestimation is difficult to quantify. Still, the risk of publication bias is higher for observational studies than for randomized controlled trials [36, 37].

## Conclusions

Our meta-analysis suggested that plasma PTX3 concentration was significantly higher in non-survivor compared to survivor patients with sepsis. Higher levels of PTX3 were significantly associated with mortality, and PTX3 was a promising biomarker for the prognosis of sepsis. Further multicenter studies with a larger sample size are warranted to verify our findings.

## Data Availability

The datasets used and/or analyzed during the current study are available from the corresponding author on reasonable request.

## Abbreviations

PTX3: Pentraxin-3
CRP: C-reactive protein
PCT: Calcitonin
HR: Hazard ratios
AUC: Area under the curve
SMD: Standard mean differences
CIs: Confidence intervals
ROC: Receiver operating characteristic
